# Supplementary material for: Dependency structure and scaling properties of financial time series are related
Source: Sci Rep. 2014 Apr 4;4:4589. doi: 10.1038/srep04589 (PMC3980463; doi:10.1038/srep04589)
Supplement: Supplementary Information — Supporting Information [file srep04589-s1.pdf]

Supplementary Material for: "Dependency structure and scaling properties of financial time series are related"

Raffaello Morales[1], T. Di Matteo[1] and T. Aste[2,3]

[1]*Department of Mathematics, King's College London, The Strand, London, WC2R 2LS, UK.*
[2]*Department of Computer Science, University College London, Gower Street, London, WC1E 6BT, UK*
[3]*Systemic Risk Centre, London School of Economics, London, WC2A 2AE, UK*

March 7, 2014

1 Data and clusters

In this section we report additional results for the most relevant clusters and industrial sectors showing dependency between multifractality and hierarchical order. In Figure 1 we show the two hierarchical structures recovered by performing DBHT clustering (top plot) and SLCA clustering (bottom plot). For the DBHT clustering one has $n \in [5, 19]$ whereas for SLCA one finds $n \in [2, 115]$. The very wide range found for SLCA seriously jeopardises the use of such clustering algorithm for the purpose of this study. Moreover, as clearly marked on the top plot in Figure 1, the DBHT identifies the clusters uniquely starting from a topological constraint, while the SLCA only provides the hierarchical organisation without clearly specifying clusters.
We report in Table 1 the Bloomberg sectors and the number of stocks they contain. The DBHT method produces thirteen clusters quite heterogeneous

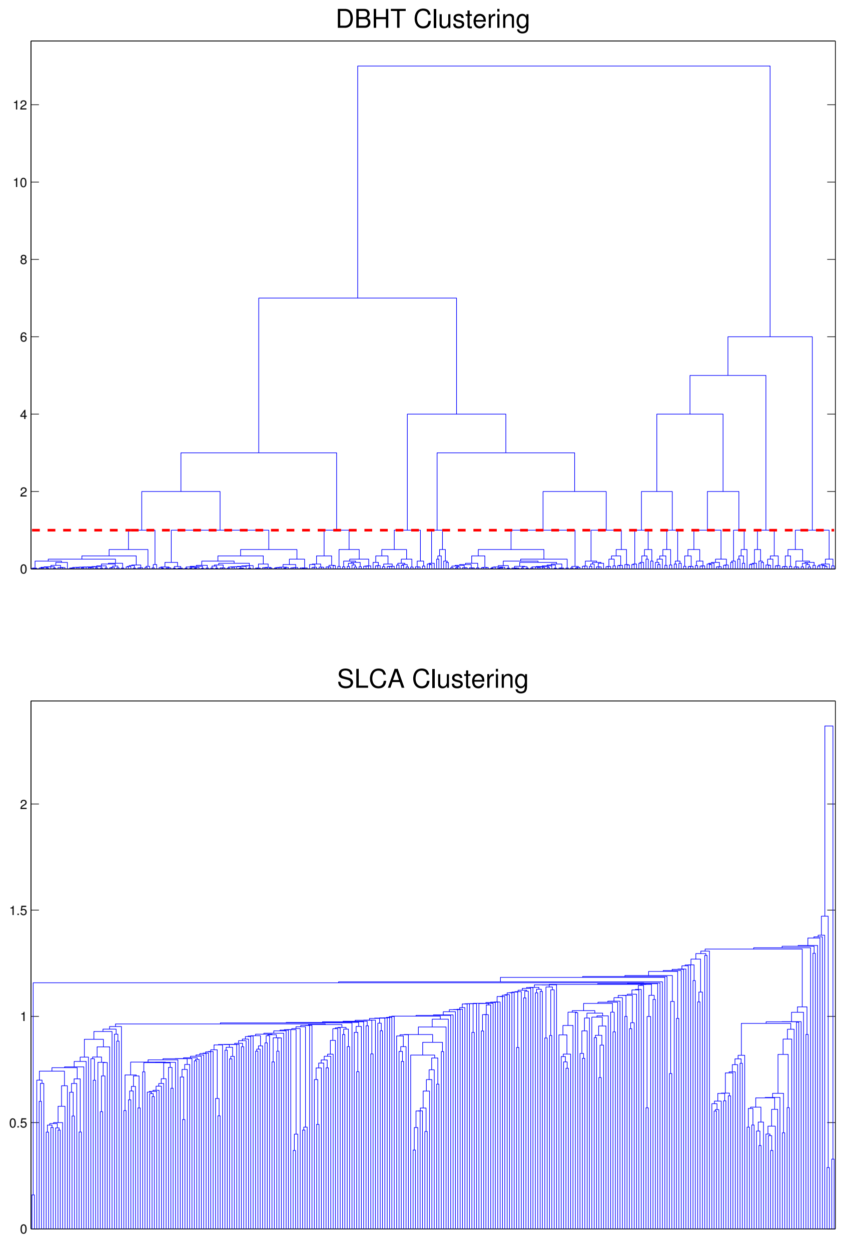

Figure 1: We show the hierarchical structures found performing DBHT clustering (top) and SLCA (bottom). The red dashed line in the top plot marks the clusters found by the DBHT. The hierarchical order ranges in $[5, 19]$ for DBHT and in $[2, 115]$ for SLCA.

Sector label	Sector	Number of stocks
1	Basic Materials	36
2	Conglomerates	7
3	Consumer Goods	48
4	Financial	50
5	Healthcare	22
6	Industrial Goods	36
7	Services	73
8	Technology	45
9	Utilities	25

Table 1: Number of stocks in each sector.

in sizes and compositions. In Table 2 we summarise the composition of the clusters in terms of stocks belonging to different sectors. We find three large clusters containing $54, 56$ and 64 stocks respectively and all quite heterogeneous in composition. Clusters labelled by 2 and 3 show a relevant fraction of stocks belonging to Financial and Industrial sectors. This is confirmed by the enrichment analysis [1], which validates the identification of the clusters with the respective sectors at a confidence level of 1%. The other clusters are significantly smaller, but still capture quite well the sector membership: cluster 13 for example contains stocks from the Utility sector only, cluster 9 and 10 from Consumer Goods and Basic Materials sectors respectively. Cluster 7 has a very large component of stocks belonging to the Technology sector, while cluster 12 is mainly composed by stocks from the Services.
For each stock we have computed the hierarchical order n, as defined in the main text. By construction the three largest clusters are the ones showing larger n on average, therefore including those stocks with more complex hierarchical structure.

Cluster	Size	Financial	Technology	Healthcare	Basic Materials	Industrial Goods	Consumer Goods	Utilities	Services	Conglomerates
1	54		16.7%	16.7%	18.5%	9.3%	**22.2%**		14.8%	1.9%
2	56	**37.5 %**	3.6%			1.8%	14.3%		**41.1%**	1.8%
3	64		6.2%	3.1%		**43.8%**	14%		**25%**	7.8%
4	10	**60%**		30%					10%	
5	20				**75%**	10%		10%	5%	
6	23	**100%**								
7	27		**96.3%**						3.7%	
8	12		**33.3%**		25%		**33.3%**		8.3%	
9	14						**100%**			
10	8				**100%**					
11	10			**80%**					20%	
12	21						4.8%		**95.2%**	
13	23							**100%**		

Table 2: **Percentage of stocks for each sector in each clusters.** We report, for each of the 13 clusters identified through the DBHT over the whole time period 2-01-1997 to 31-12-2012, the percentages of the stocks from each of the 9 Bloomberg sectors.

The top plot in Figure 2 shows multifractality plotted versus hierarchical order for stocks belonging to the sector of Basic Materials. We can see a very fast growth of multifractality for hierarchical orders in the range $[7, 12]$, followed by something like a flat trend for higher orders. This behaviour suggests that the dependence existing between the two variables may most probably be non-linear (see the figure captions for quantitative details on the trends). The same inflection suggesting non-linear behaviour is also observed on stocks belonging to the Utilities sector (see bottom plot in Figure 2). These observations seem to hint at a saturation of multifractality beyond a certain order, as if the effect of the many risks associated with the dendrogram nodes would decrease with the order of the node.

We have also looked at the trends observed on sub clusters, i.e. groups of stocks below the cluster level in the hierarchy. Restricting to hierarchical levels below the clusters has two opposite consequences: on the one hand it allows to be more strict in factoring out a larger number of common hierarchical levels, hence making the differentiation between different stocks more relevant. On the contrary, it can reduce significantly the number of stocks and thus potentially increase the influence of noise on the trends. After a thorough analysis of all sub clusters of different order, we found that in all cases the trends recovered on sub clusters are, when significant, the same observed on the clusters. We use the notation $\mathcal{S}^j_{i,k}$ to label such sets, where $j = 1, \ldots, 13$ denotes the cluster label, i denotes the order below the cluster and $k = 1, \ldots, 2^i$ denotes which of the 2^i sub clusters at each order we are considering. We plot in Figure 3 $\Delta H(1, 2)$ versus n for cluster no.6 in Table 1 in the main article and for two sub clusters $\mathcal{S}^3_{1,1}$ and $\mathcal{S}^2_{2,1}$. The sub cluster $\mathcal{S}^3_{1,1}$ is very similar to cluster no.3 shown in the main text: cluster no.3 in fact results from the merging of $\mathcal{S}^3_{1,1}$ and $\mathcal{S}^3_{1,2}$, that contains only 5 stocks.

2 A different data set: London Stock exchange

The main empirical result reported in this paper has been verified also on a different data set. We have analysed the set of 185 most capitalised stocks traded at the London Stock Exchange (LSE) in the period 04/01/2000-21/08/2013. Data has been provided by Bloomberg. The DBHT returned a range of hierarchical order comparable to that obtained for the NYSE, with stocks exhibiting $n \in [4, 17]$. We plot in Figure 4 the equivalent of Figure 2 in the main text. We observed the same increasing trend up to n=12,

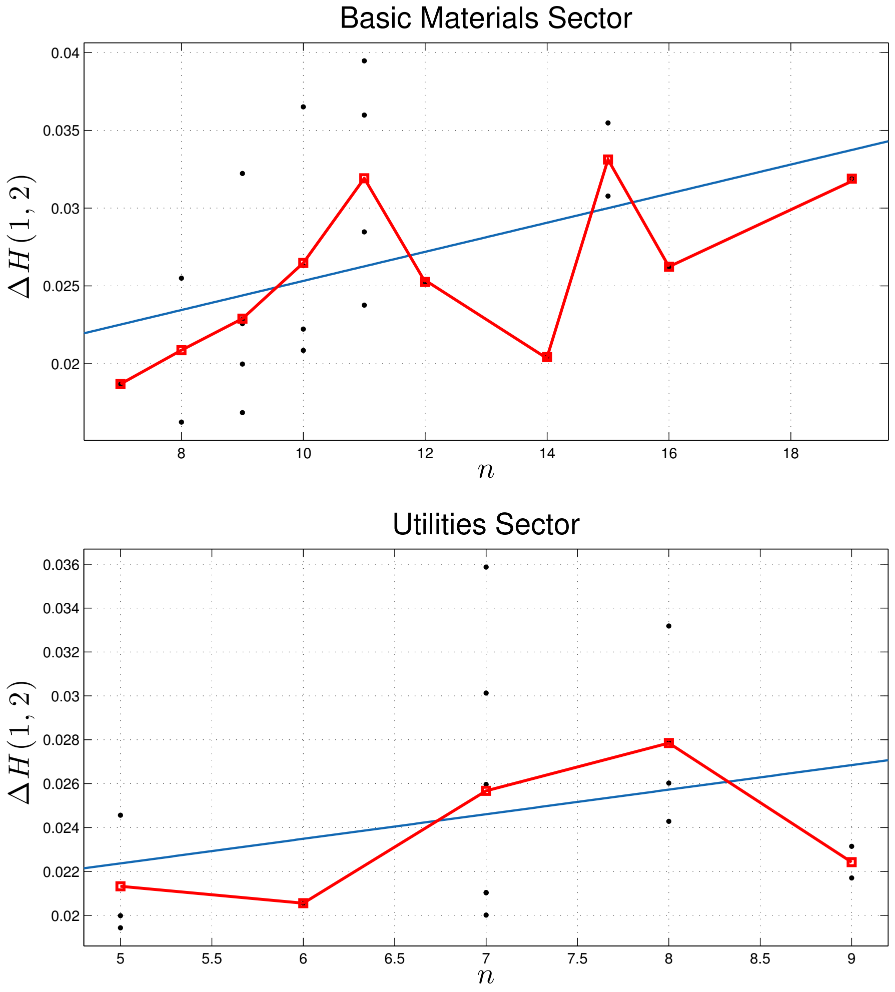

Figure 2: We plot in black dots $\Delta H(1,2)$ against the hierarchical order n for stocks in the Basic Materials sector (top) and in the Utilities sector (bottom). In both plots the blue line is the best fit of the dots, while the red squares are the averages of $\Delta H(1,2)$ for each fixed order. Correlation coefficients and corresponding p-Values are 0.41 and 0.04 for Basic Materials and 0.29 and 0.27 for Utilities.

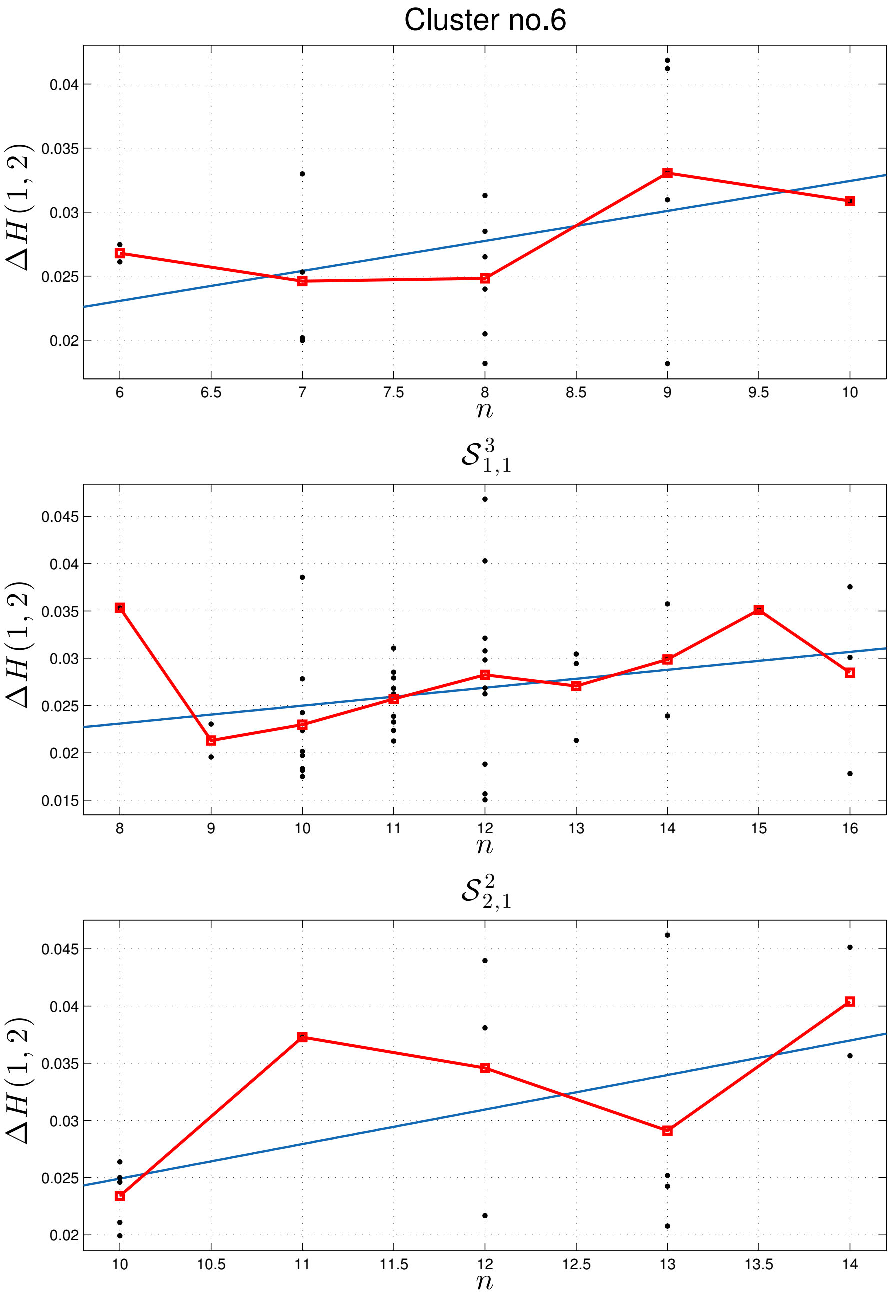

Figure 3: Black dots are $\Delta H(1,2)$ against the hierarchical order n for stocks in cluster no.6 (top) and in the sub clusters $\mathcal{S}^3_{1,1}$ (middle) and $\mathcal{S}^2_{2,1}$ (bottom). In all three plots the blue line is the best fit of the dots, while the red squares are the averages of $\Delta H(1,2)$ for each fixed order. Correlation coefficients and corresponding p-Values are: 0.16 and 0.4 for Cluster 6, 0.25 and 0.01 for $\mathcal{S}^3_{1,1}$ and 0.63 and 0.25 for $\mathcal{S}^2_{2,1}$.

followed by a drop and a plateau at higher order, where the observations are however fewer. The regime $n \in [4, 12]$ confirms the observation reported in the main text for NYSE: within a certain range of orders, multifractality and hierarchical order appear to be positively correlated.

3 Asian markets: one dominant market mode

We have also analysed the set of 386 most capitalised stocks listed in the Tokyo Stock Exchange (TSE) and the 136 most capitalised stocks in the Hong Kong Stock Exchange (HKSE) in the period 01/01/2003-26/08/2013. For both markets, the DBHT returns a taxonomy of clusters very different from that found in the NYSE and LSE data. For the Japanese market we found only six clusters, among which there is a gigantic cluster containing 300 stocks, while the remaining stocks are more or less uniformly distributed in the remaining clusters. A closer inspection of the correlation matrix of this system reveals that, on average, these stocks show correlation larger than those observed in the western markets, with an average correlation of 0.38 (compared to 0.18 found in NYSE and 0.20 for LSE). As a result of this very homogeneous taxonomy of clusters, stocks exhibit hierarchical order varying in the range $[3, 121]$: as discussed in the main text, a dendrogram with a small number of clusters necessary entails the hierarchy to develop vertically. For the HKSE we found six clusters and $n \in [4, 51]$. It is nearly impossible to distinguish the effect of such a wide range of orders on the multifractal properties of stocks, but nonetheless the interdependence between the two variables $\Delta H(1, 2)$ and n is still observed for small n (see Figure 5 (top) for TSE and Figure 5 (bottom) for HKSE). We mention that the number of observations for each n is very small to allow any robust statistical conclusion (the errors are consequently very large).

To remove the presence of these very large hubs of correlated stocks, we have performed a detrending of the time series in order to get rid of the dominant market mode and thus observe a more heterogeneous structure of clusters. Returns are considered to follow a one factor linear model of the following form [2]

$$r_{i,t} = a_i + \beta_i I_t + c_{i,t} \tag{1}$$

where $I_t = 1/N \sum_{i=1}^{N} r_{i,t}$ is the composite index with homogeneous weights. After estimating the coefficients β_i for each stock, the correlation matrix has been computed on the residuals $c_{i,t}$, thus deprived of any common trend.

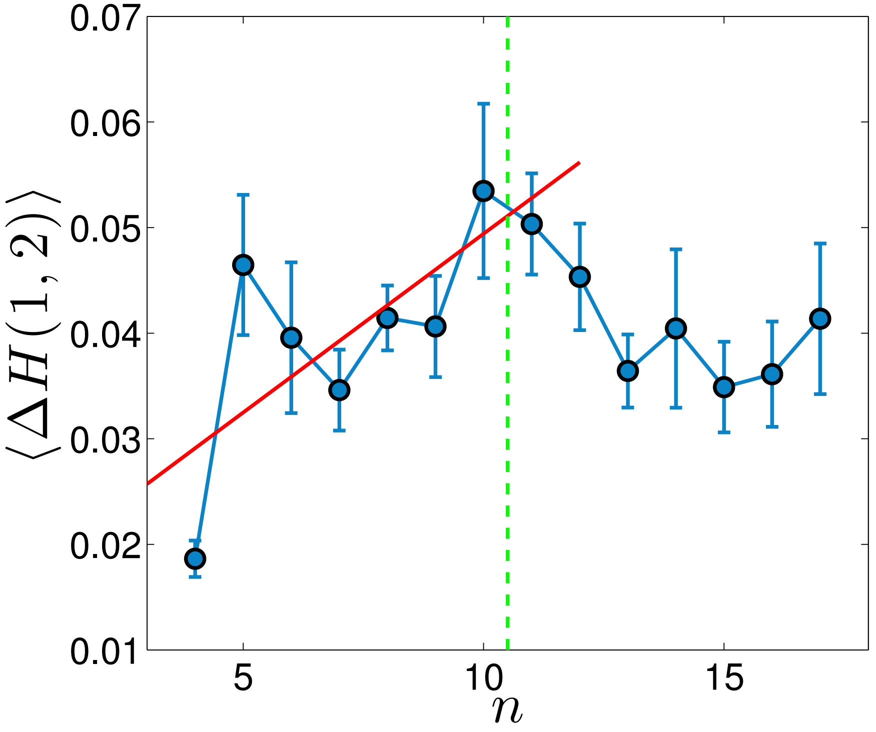

Figure 4: Positive correlation between multifractality and hierarchical order for stocks traded in LSE. We plot in blue circles the average $\langle \Delta H(1,2) \rangle$ for each observed hierarchical order against the corresponding n. Error bars are standard errors on the mean, computed as $s/\sqrt{N_n}$, with N_n the number of stocks having order n and s the standard deviation on the mean. The red line is the best linear fit on the averages and the vertical dashed green line marks the end of the linear trend.

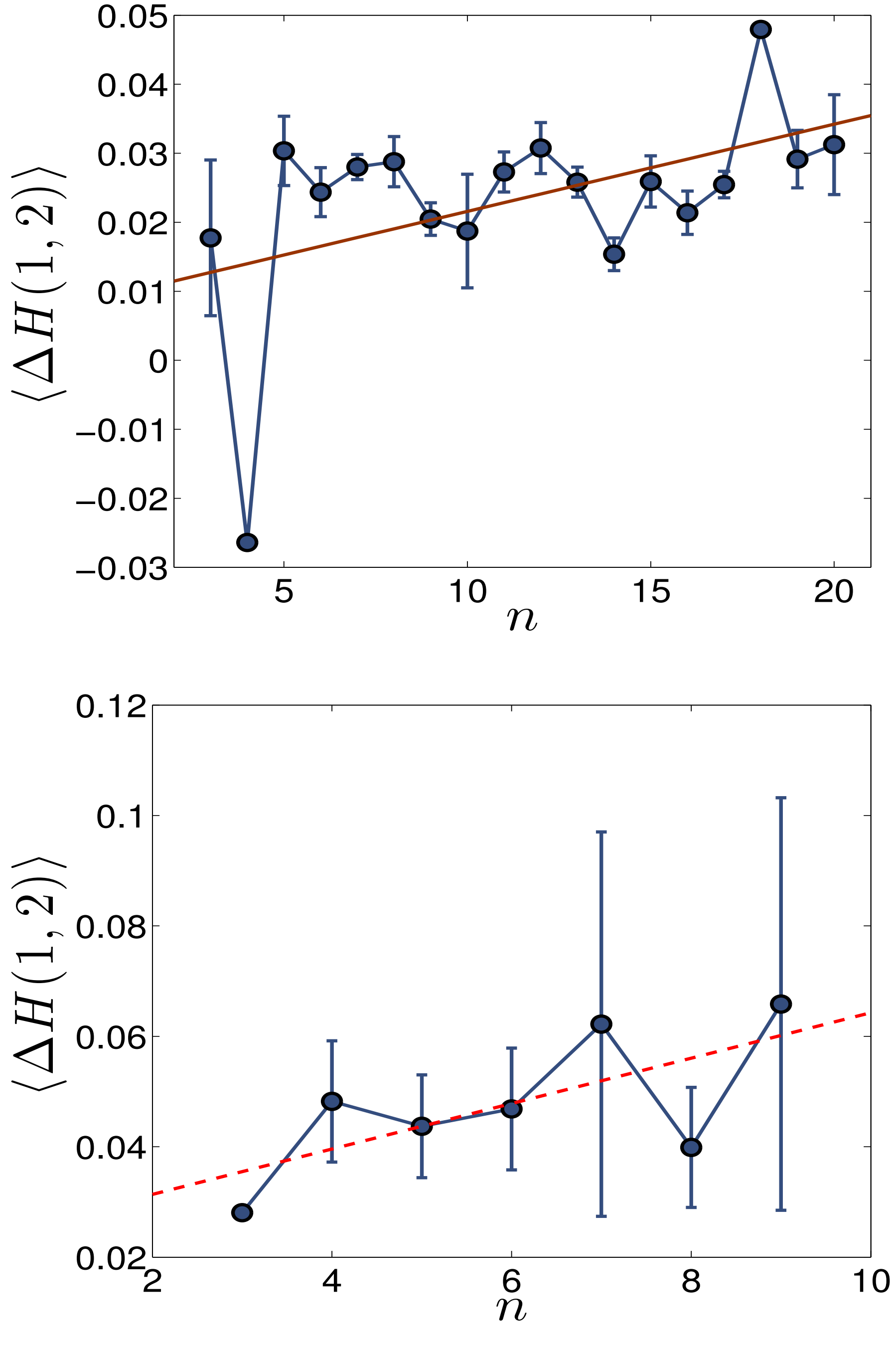

Figure 5: (Top) Relationship between $\langle \Delta H(1,2) \rangle$ and n for $n \in [3, 20]$, neglecting all higher hierarchical orders ($n \in [21, 121]$) for TSE. (Bottom) Relationship between $\langle \Delta H(1,2) \rangle$ and n for $n \in [3, 9]$, neglecting all higher hierarchical orders ($n \in [10, 51]$) for HKSE. The solid lines are the best fits on the averages. In both cases a positive trend is observed. Correlation coefficients and corresponding p-Values are 0.41 and 0.09 for TSE and 0.61 and 0.14 for HKSE.

As expected, after detrending the number of clusters increases to 21 and the range of n is found to be $[5, 21]$. However, multifractality appears to be completely uncorrelated with n, as can be seen from the (almost) flat trend in Figure 6. This observation has in our opinion the following interpretation: in a very correlated data set, the market mode is definitely the most relevant factor affecting the dynamics of the stocks. As a consequence, removing the common trend all but removes the most relevant source of complexity of the time series, whose remaining heterogenous properties cannot explain the correlation between hierarchical order and multifractality. Note that this interpretation is not at odds with what postulated in our model, where one single market mode is equivalent to a vanishing hierarchy where the intermediate nodes do not introduce any diversification in the market as all stocks share the same set of nodes. The Japanese market thus appears to be much less diversified than the American and British ones and one factor seems to be enough to describe its dynamics. In general, it remains to be ascertain whether the DBHT is always the correct tool to measure the hierarchy in a very correlated data set, where very large hubs of stocks tend to appear, thwarting the possibility of tracking the effect of the hierarchy on the multi-scaling properties of the time series. In markets diversified enough though, like the NYSE, the effect of many risk factors is still reasonably well detected by the DBHT.

4 Bootstrapping the DBHT

Although the DBHT is a deterministic algorithm, which means that repeating the clustering many times on the same dataset yields the same result, the correlation matrix is inherently noisy and small perturbations of its entries may have dramatic fallouts on the topology of the hierarchical structure. In particular, even slight modifications in the ranking of correlations are likely to undermine the organisation recovered with the original configuration. For this reason it makes sense to study the robustness of the DBHT method by validating the dendrogram obtained against some statistical significance level and, to this end, bootstrapping is probably the most suitable tool [3, 4]. The estimates whose accuracy need to be validated are the hierarchical orders of the stocks. We proceed as follow: we construct a number of synthetic resamples of the original dataset, where each time the surrogate data are obtained by the usual procedure of sampling with replacement. Each time

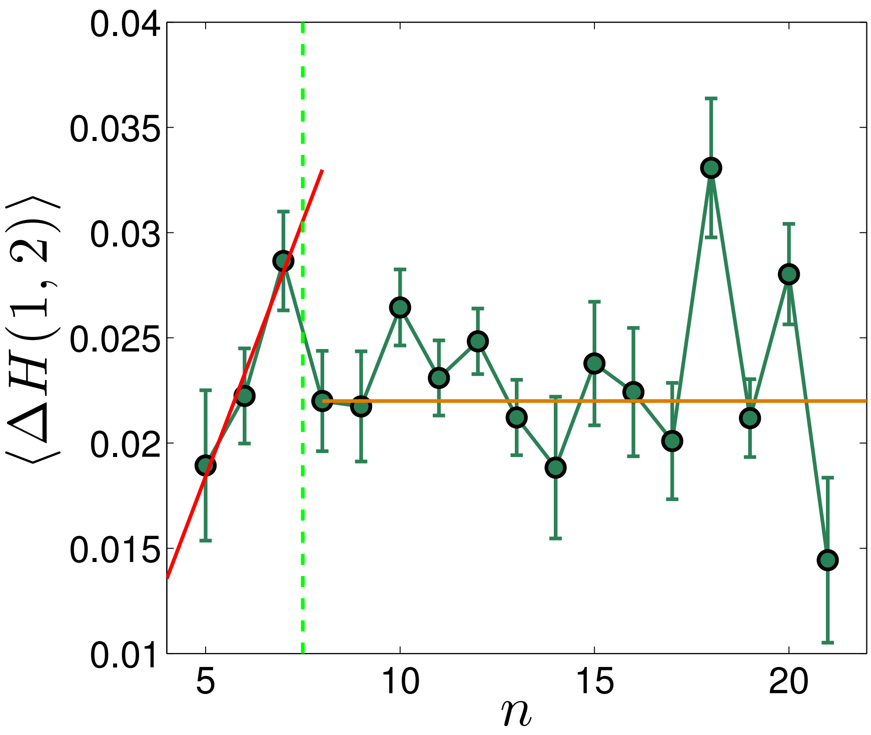

Figure 6: Correlation between multifractality and hierarchical order after detrending for stocks traded in TSE. We plot in green circles the average $\langle \Delta H(1,2) \rangle$ for each observed hierarchical order against the corresponding n. Error bars are standard errors on the mean, computed as $s/\sqrt{N_n}$, with N_n the number of stocks having order n and s the standard deviation on the mean. The red line is the best linear fit on the averages for $n \in [5, 7]$, while the orange line is the fit for $n \in [8, 21]$. The dashed line marks the end of the initial increasing trend.

the statistics of interest is computed and at the end the original observation is validated against the distribution of the resample estimates obtained from the surrogate series. Since we are interested in giving accurate estimates of the orders of all stocks, the DBHT is performed on each bootstrapped correlation matrix for a total of 1000 resamples. From each of these dendrograms constructed from surrogate correlation matrices we extract the vector of bootstrapped hierarchical orders $\mathbf{n}_\beta = (n^1, n^2, \ldots, n^N)_\beta$, where $N = 342$ is the number of stocks in the databset and $\beta = 1, \ldots, 1000$. Then we compare the order vector $\hat{\mathbf{n}}$ observed on the empirical time series with the synthetic distribution of bootstrapped values. For each stock, we discard the empirical order as non-reliable if it falls beyond the two-sided 0.9 level of probability obtained from the cumulative distribution function of the bootstrapped series. As an example of the validation procedure, we report the case of the order of the stock Air Products and Chemicals INC in Figure 7: the order $\hat{n}$ measured on the original DBHT is not validated by the bootstrap test.

The number of stocks which fail the bootstrap test is remarkably high and, by coincidence, is exactly half of the total number of stocks. We show in Figure 8 the distribution of the measured order before the bootstrapping (blue bars) and the distribution of the order of the stocks validated (dashed red line). Although the number of stocks is halved, the shape of the distribution remains similar. We then denote the set of valid hierarchical orders as n_B.

After the bootstrap validation, the trends recovered in the plots of $\Delta H(1,2)$ versus n reveal some further hidden structure. We show in Figure 9 the trends observed on clusters 2, 3 and sub cluster $\mathcal{S}^{3,B}_{1,1}$, where the stocks shown are only those that have been validated via bootstrapping. As also shown in Figure 8, the stocks excluded are more or less uniform in their order, which results in stocks with very large (above 15) and very small (below 7) order to almost disappear from the set. Although the number of stocks is reduced, the dependence of multifractality on the hierarchical order is seen even more neatly. Moreover the non-linear behaviour guessed from the plots presented in the previous section is now even more evident from the middle plot in Figure 9.

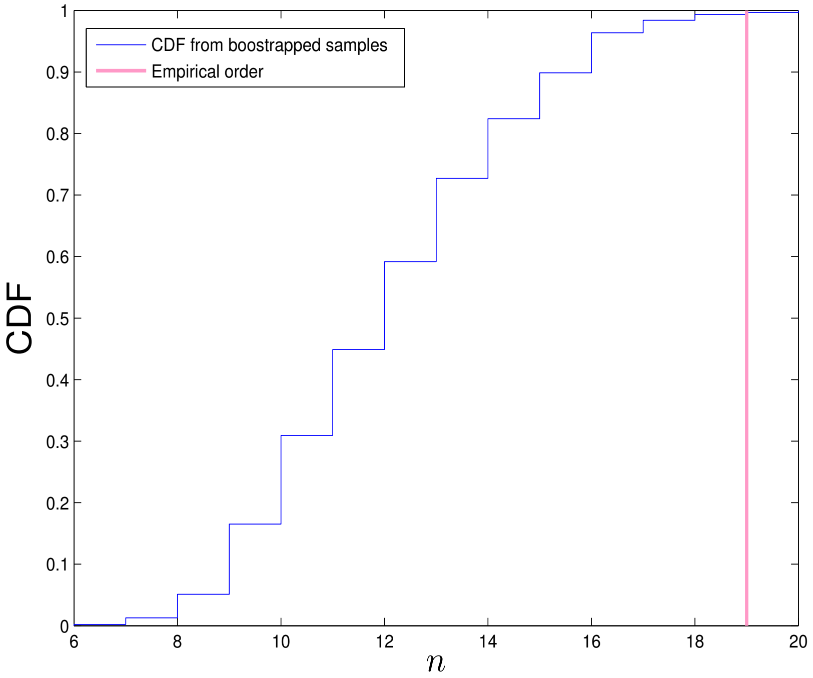

Figure 7: We plot (blue line) the cumulative distribution function of the bootstrapped hierarchical order for the stock Air Products and Chemicals INC. The pink vertical line corresponds to the hierarchical order of the same stock obtained from the DBHT on the original dataset. Its level of probability is such that the estimated order cannot be validated.

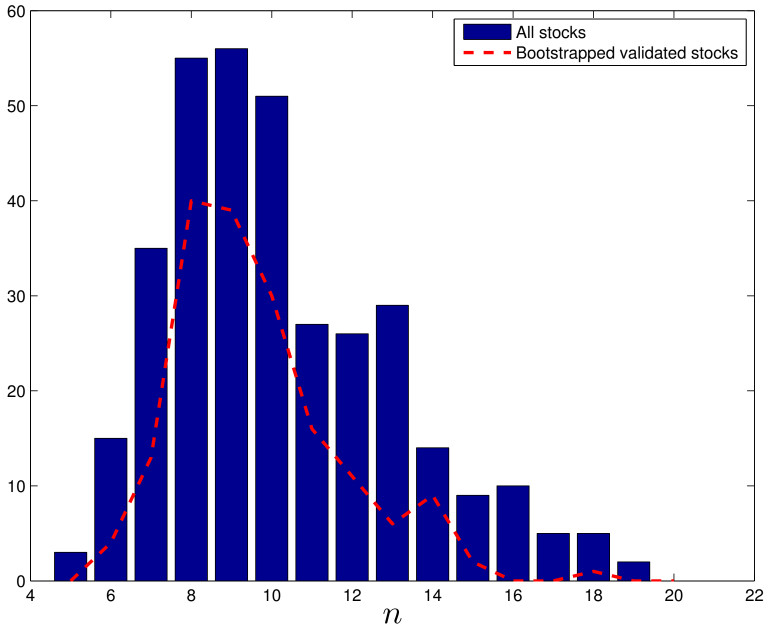

Figure 8: We plot in blue bars the histogram of the hierarchical orders obtained from the DBHT for the complete dataset. The thick dashed red line is the histogram of the hierarchical orders for those stocks that passed the bootstrapping validation.

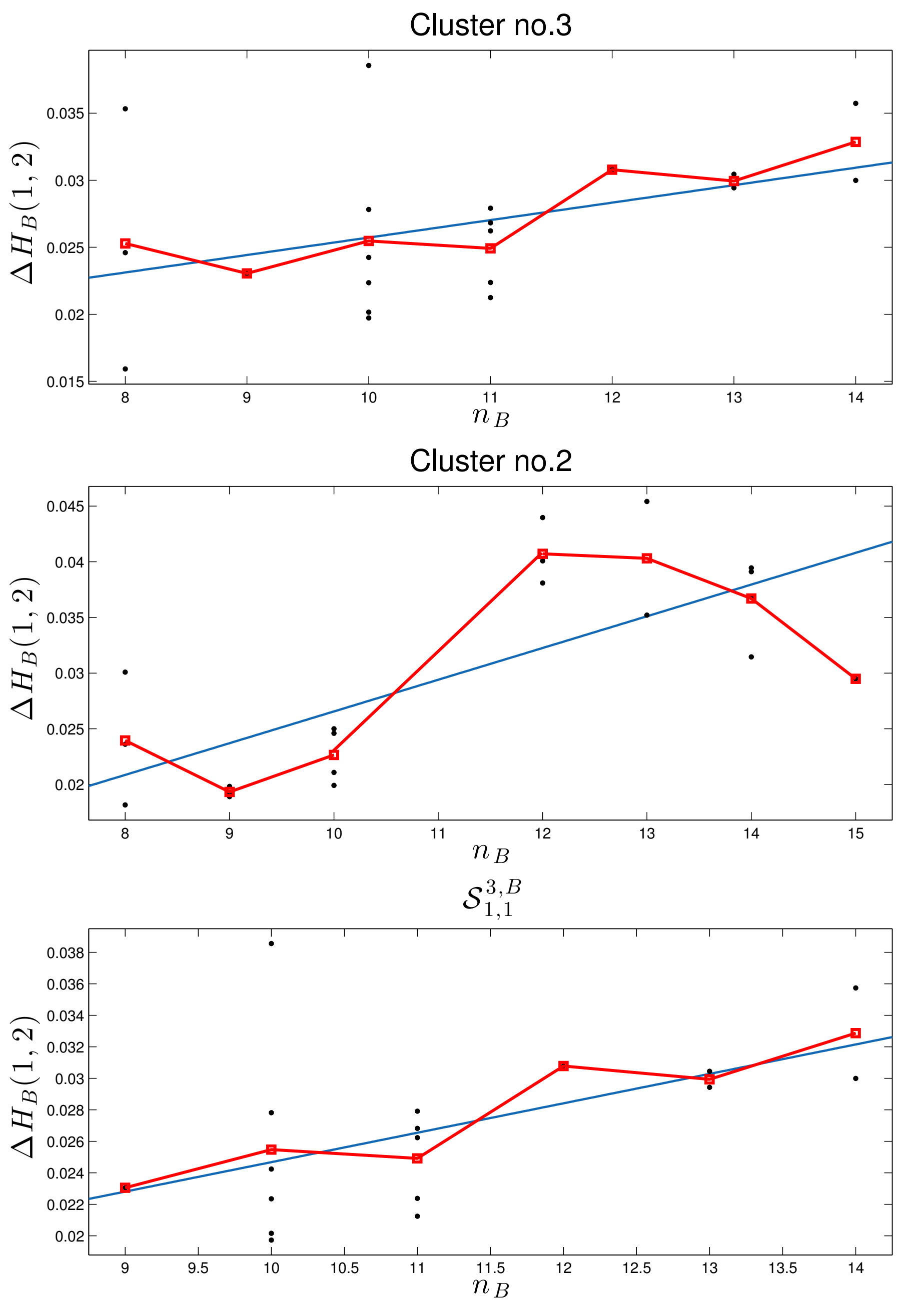

Figure 9: $\Delta H(1,2)$ against the hierarchical order n for stocks in cluster no.3 (top), cluster no.2 (middle) and sub cluster $\mathcal{S}_{1,1}^{3,B}$ (bottom) for stocks whose order has been validated via bootstrapping. In all three plots the blue line is the best fit of the dots, while the red squares are the averages of $\Delta H(1,2)$ for each fixed order.

5 Time-varying hierarchical structure and multifractal properties

In this section we report more details about the time varying hierarchical structure measured on a reduced set of empirical data, which is used to simulate the DHM in the main text of the article. We chose the following 25 stocks from the set (we report the tickers in parenthesis) : (1) First Horizon National Corp (FHN), (2) Abbot Laboratories (ABT), (3) Adobe Systems (ADBE), (4) Advanced Micro Devices INC (AMD), (5) Aflac INC (AFL), (6) Air Products and Chemicals INC (APD), (7) Allergan INC/United States (AGN), (8) Honeywell International/United States (HON), (9) The Allstate Corporation (ALL), (10) Altera Corp (ALTR), (11) Alcoa INC (AA), (12) Barrick Gold Corp (ABX), (13) American Elettric Power CO INC (AEP), (14) American Express Corp (AXP), (15) American Greetings (AM), (16) Amgen INC (AMGN), (17) Hess Corp (HES), (18) American International Group INC (AIG), (19) Anadarko Petroleum Corp (APG), (20) Analog Devices (ADI), (21) Aon Corp (AOC), (22) Apache Corp (APA), (23) Apple INC (AAPL), (24) Applied Materials INC (AMAT), (25) Archer-Daniels-Midlan Co(ADM). The choice was arbitrary and also other choices have been explored without revealing any blatant bias or evident difference. We first show in Figure 10 the hierarchical structure detected on the whole period 1/1/1995-22/10/2012. We have split the time series into two tranches of length 2013 days: the first one spanning the period 1/1/1995 through 31/12/2004, the second one 31/12/2004 through 22/10/2012. The DBHT has been then performed on both tranches separately, after having computed the two weighted correlation matrices $\hat{C}_{w,T_1}$ and $\hat{C}_{w,T_2}$ [5]. We show the two hierarchical structures $\mathcal{H}_{T_1}$ and $\mathcal{H}_{T_2}$ obtained over the two different time periods in Figure 11. These two hierarchical structures have been then used to simulate the model producing the results reported in the main text. To each stock $i = 1, \ldots, 25$ is associated a hierarchical tree $\Gamma_i^{T_k}$, for $k = 1, 2$, including all the nodes above the stock. We plot in Figure 12 the simulated returns process and volatility process for the stock labelled 11 in Figure 11, as an example of the effect of the changing hierarchy. In the tranche T_1 this particular stock shares one common risk with stock labelled 14, then one common risk with the pair of stocks $6, 15$ and so on. In the tranche T_2 it shares one common risk with stock 25, one common risk with the pair $3, 9$ and so on. Overall the topology of the dendrogram changes quite dramati-

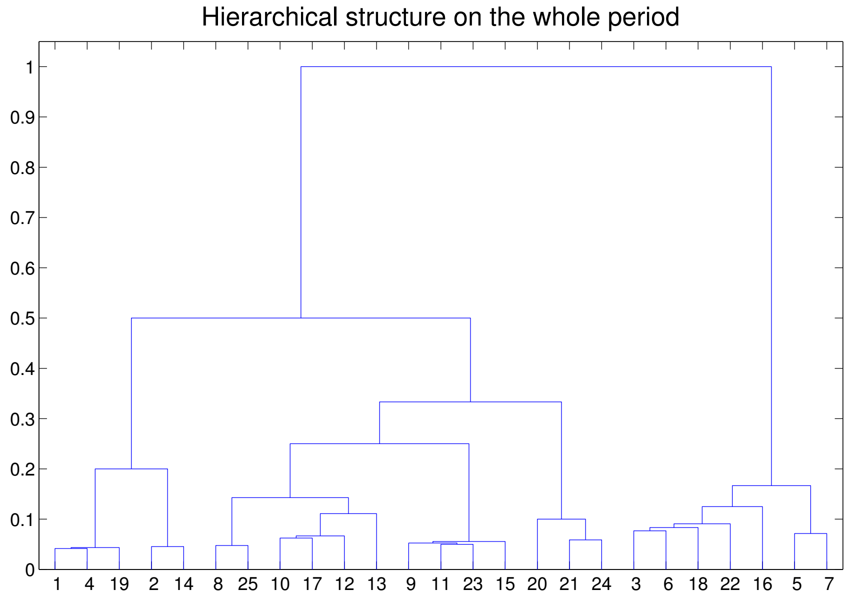

Figure 10: The hierarchical structure of the set of 25 stocks obtained through the DBHT clustering algorithm on the time period 1/1/1995-22/10/2012.

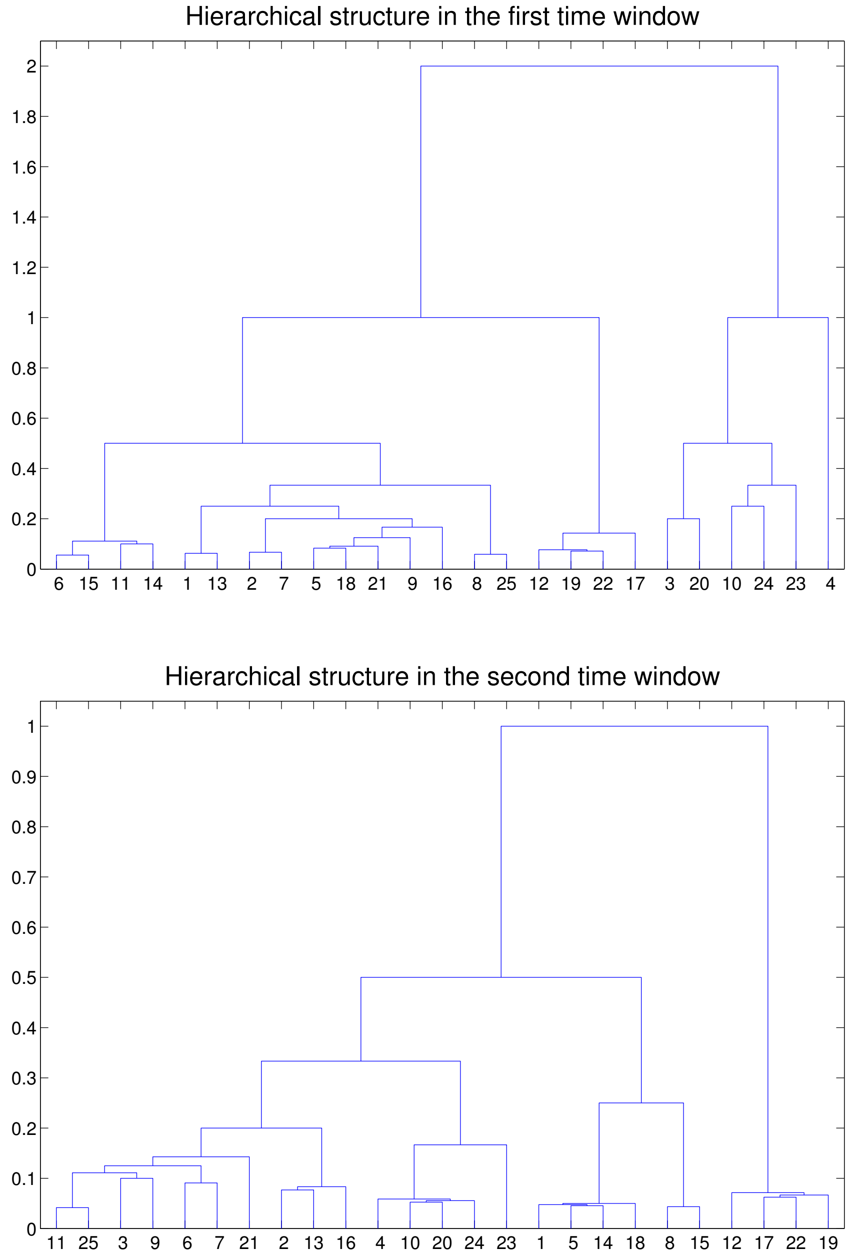

Figure 11: We show the two hierarchical structures obtained via the DBHT on two separate time windows: the first one (top) spanning the period 1/1/1995 through 31/12/2004, the second one (bottom) 31/12/2004 through 22/10/2012.

	Increasing order		Decreasing order	
	T_1	T_2	T_1	T_2
H(1)	0.58	0.62	0.61	0.60
H(2)	0.48	0.51	0.48	0.51
$\Delta H(1,2)$	0.10	0.12	0.13	0.09

Table 3: Scaling exponents $H(1), H(2)$ and $\Delta H(1,2)$ obtained as averages with error over 1000 realisations of DHM with the two regimes hierarchical structures: $\mathcal{H}_{T_1}$ on the tranche T_1 and $\mathcal{H}_{T_2}$ on the tranche T_2. The probabilities of the risks have been chosen to be uniformly distributed in $[0.4, 0.6]$. Standard errors are of order 10^{-3} and not reported. All mean values in T_1 have been statistically checked to be significantly different from those in T_2 via a t-test, returning p-value always negligible at a threshold of 5% confidence level.

cally in the second tranche and this result in a rather different spreading of the risks. For the stock labelled 11 the number of risks increase from 5 to 8. One can clearly see the effect of the larger number of risks which cause fluctuations to become much larger.
We performed an extensive analysis studying the measured multifractality and scaling exponents on many synthetic time series, considering separately those whose hierarchical order increases from those whose hierarchical order decreases. We have simulated 1000 realisations of a 25 stocks 2-regimes multivariate DHM of length 4000 with hierarchical structures given by $\mathcal{H}_{T_1}$ and $\mathcal{H}_{T_2}$ on the two tranches T_1 ($t \in [1, 2000]$) and T_2 ($t \in [2001, 4000]$) respectively. Each time we computed the scaling exponents and multifractality indicator $\Delta H(1,2)$, keeping track of whether, for each stock i, $n_{T_1} > n_{T_2}$ or $n_{T_1} < n_{T_2}$. For the hierarchies in figures 11 we find 15 stocks whose order increases and 10 stocks whose order decreases. With this analysis one can test whether there is a significant correlation between the hierarchical order and the scaling properties of the synthetic time series. We performed this analysis for different values of the probabilities of the risks considering all p's to be uniformly distributed in $[0.4, 0.6]$, $[0.6, 0.8]$ and $[0.8, 1]$ respectively. The three sets of results are shown in Tables 3, 4 and 5. From these tables two general facts stand out:

- the observed multifractality is correlated with the order of the hierarchy. In all cases indeed we observed higher multifractality on the

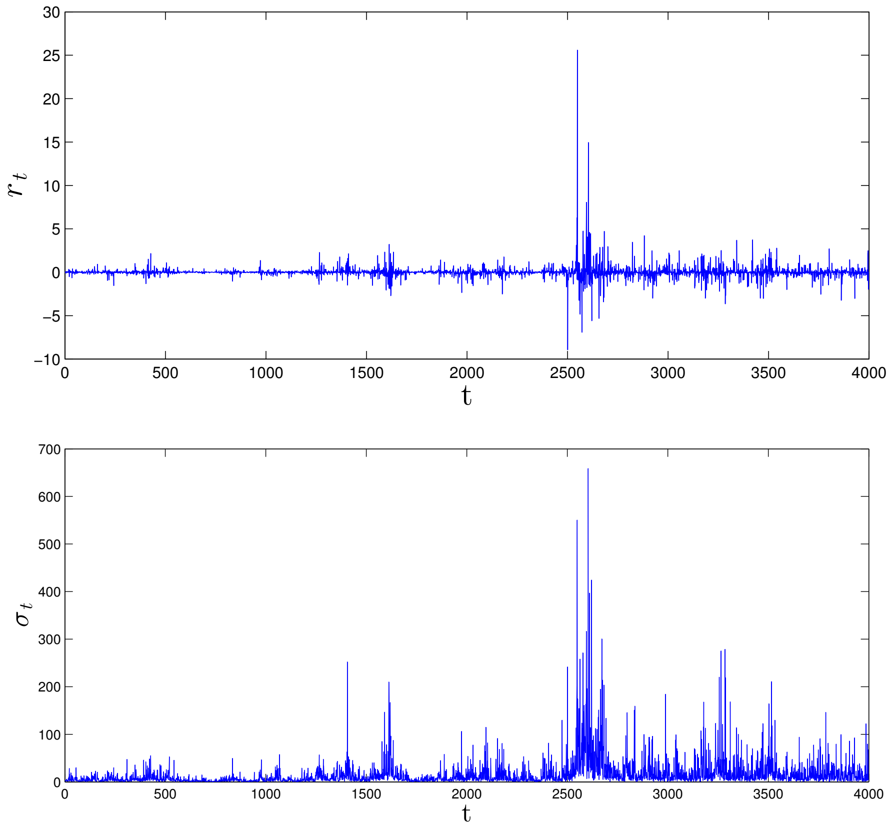

Figure 12: We plot returns (top) and volatility (bottom) for the simulated process whose hierarchical structure is $\Gamma_{11} = \Gamma_{11}^{T_1}$ for $t \in [1, 2013]$ and $\Gamma_{11} = \Gamma_{11}^{T_2}$ for $t \in [2014, 4026]$. The common volatility process x_t has been simulated as discussed in the Methods section of the main text with parameters $\lambda = 0.2$ and $T = 800$.

	Increasing order		Decreasing order	
	T_1	T_2	T_1	T_2
H(1)	0.55	0.60	0.58	0.57
H(2)	0.48	0.51	0.48	0.51
$\Delta H(1,2)$	0.07	0.09	0.10	0.06

Table 4: Scaling exponents $H(1), H(2)$ and $\Delta H(1,2)$ obtained as averages with error over 1000 realisations of DHM with the two regimes hierarchical structures: $\mathcal{H}_{T_1}$ on the tranche T_1 and $\mathcal{H}_{T_2}$ on the tranche T_2. The probabilities of the risks have been chosen to be uniformly distributed in $[0.6, 0.8]$. Standard errors are of order 10^{-3} and not reported.

	Increasing order		Decreasing order	
	T_1	T_2	T_1	T_2
H(1)	0.53	0.56	0.54	0.55
H(2)	0.48	0.51	0.48	0.51
$\Delta H(1,2)$	0.05	0.05	0.06	0.04

Table 5: Scaling exponents $H(1), H(2)$ and $\Delta H(1,2)$ obtained as averages with error over 1000 realisations of DHM with the two regimes hierarchical structures: $\mathcal{H}_{T_1}$ on the tranche T_1 and $\mathcal{H}_{T_2}$ on the tranche T_2. The probabilities of the risks have been chosen to be uniformly distributed in $[0.8, 1]$. Standard errors are of order 10^{-3} and not reported.

tranches where the hierarchical order was larger than in those where it was smaller. As one can read off all tables, simulated time series whose order increase switching from the first to the second hierarchy show increasing multifractality, whereas series whose order decreases show decreasing multifractality.

- Multifractality is largest for parameters p's in $[0.4, 0.6]$ and decreases significantly in the other two sets of simulations. The increase or decrease of multifractality and scaling exponents $H(1)$ and $H(2)$ have been statistically validated through t-test at two-sided 5% confidence level: the p-values are always extremely small, confirming a true differentiation between the scaling properties in T_1 and T_2.

Among the 25 stocks analysed there are 4 stocks whose hierarchical order $n_{T_1} = n_{T_2}$. We show in Figure 13 the distribution of $\Delta H(1,2)$ obtained on the two different tranches for these 4 stocks. Compared to the plots in Figure 7 in the main text, we can see how the difference between the values obtained in the two windows is much smaller for the four stocks with $n_{T_1} = n_{T_2}$. This tells us that keeping the hierarchy constant has not a sizeable impact on the multifractal properties of the stocks.
We also report in Table 6 and 7 the numerical values of the quantiles shown in Figure 7 in the main text. Table 6 shows quantiles for the stocks having $n_{T_1} < n_{T_2}$ while Table 7 shows quantiles for stocks having $n_{T_1} > n_{T_2}$. Finally, quantiles for stocks having $n_{T_1} = n_{T_2}$ are reported in Table 8.

6 Model

6.1 Proof of equation (4)

Let us consider two return processes $r_{i,t}$ and $r_{j,t}$ having arbitrary hierarchical trees:

$$\Gamma_i = \{a_1, \ldots, a_{n_i}\} \tag{2}$$
$$\Gamma_j = \{b_1, \ldots, b_{n_j}\} \tag{3}$$

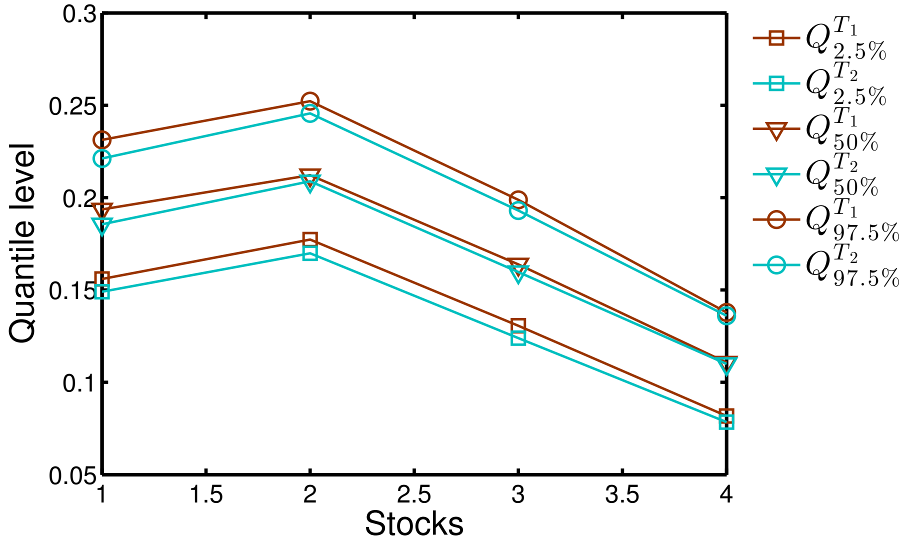

Figure 13: The plot shows the q-quantiles $Q_q^{T_1}$ and $Q_q^{T_2}$ for $q = \{2.5\%, 50\%, 97.5\%\}$ for the 4 DHM simulated time series whose hierarchical order $n_{T_1} = n_{T_2}$. Red lines correspond to p-quantiles in T_1 while light blue lines to p-quantiles in T_2.

Increasing order	
T_1	T_2
$[Q^{T_1}_{2.5\%}, Q^{T_1}_{50\%}, Q^{T_1}_{97.5\%}]$	$[Q^{T_2}_{2.5\%}, Q^{T_2}_{50\%}, Q^{T_2}_{97.5\%}]$
$[0.0814, 0.1095, 0.1362]$	$[0.1706, 0.2101, 0.2499]$
$[0.0188, 0.0470, 0.0683]$	$[0.1077, 0.1405, 0.1697]$
$[0.0998, 0.1326, 0.1632]$	$[0.1507, 0.1887, 0.2244]$
$[0.1033, 0.1365, 0.1664]$	$[0.1530, 0.1911, 0.2297]$
$[0.1033, 0.1358, 0.1657]$	$[0.1647, 0.2040, 0.2428]$
$[0.1052, 0.1351, 0.1630]$	$[0.1317, 0.1651, 0.1964]$
$[0.0498, 0.0790, 0.1031]$	$[0.0804, 0.1101, 0.1365]$
$[0.0809, 0.1097, 0.1370]$	$[0.1551, 0.1905, 0.2256]$
$[0.1073, 0.1371, 0.1681]$	$[0.1298, 0.1644, 0.2006]$
$[0.1024, 0.1363, 0.1650]$	$[0.1706, 0.2053, 0.2433]$

Table 6: In this table we report the set of quantiles obtained from the distribution of the multifractality proxy $\Delta H(1,2)$ on the two tranches T_1 and T_2 for 1000 simulated time series whose hierarchical order $n_{T_1} < n_{T_2}$. Probabilities p's of the model are initialised to take values in $[0.4, 0.6]$. One observes an almost rigid shift towards larger values of the distribution of the observed $\Delta H(1,2)$ when the order increases.

Decreasing order	
T_1	T_2
$[Q^{T_1}_{2.5\%}, Q^{T_1}_{50\%}, Q^{T_1}_{97.5\%}]$	$[Q^{T_2}_{2.5\%}, Q^{T_2}_{50\%}, Q^{T_2}_{97.5\%}]$
$[0.1304, 0.1628, 0.1950]$	$[0.1126, 0.1446, 0.1760]$
$[0.1564, 0.1941, 0.2327]$	$[0.1251, 0.1608, 0.1933]$
$[0.2061, 0.2484, 0.2893]$	$[0.1313, 0.1651, 0.1993]$
$[0.1064, 0.1375, 0.1673]$	$[0.0792, 0.1079, 0.1332]$
$[0.0852, 0.1124, 0.1411]$	$[0.0257, 0.0517, 0.0736]$
$[0.1002, 0.1328, 0.1624]$	$[0.0778, 0.1086, 0.1343]$
$[0.1514, 0.1902, 0.2253]$	$[0.1051, 0.1348, 0.1642]$
$[0.2086, 0.2471, 0.2954]$	$[0.0847, 0.1127, 0.1403]$
$[0.1093, 0.1425, 0.1721]$	$[0.0517, 0.0789, 0.1016]$
$[0.1913, 0.2222, 0.2743]$	$[0.1042, 0.1359, 0.1629]$
$[0.1106, 0.1427, 0.1759]$	$[0.0827, 0.1113, 0.1387]$

Table 7: In this table we report the set of quantiles obtained from the distribution of the multifractality proxy $\Delta H(1, 2)$ on the two tranches T_1 and T_2 for 1000 simulated time series whose hierarchical order $n_{T_1} > n_{T_2}$. Probabilities p's of the model are initialised to take values in $[0.4, 0.6]$. One observes an almost rigid shift towards smaller values of the distribution of the observed $\Delta H(1, 2)$ when the order decreases.

Same order	
T_1	T_2
$[Q^{T_1}_{2.5\%}, Q^{T_1}_{50\%}, Q^{T_1}_{97.5\%}]$	$[Q^{T_2}_{2.5\%}, Q^{T_2}_{50\%}, Q^{T_2}_{97.5\%}]$
$[0.1559, 0.1936, 0.2312]$	$[0.1490, 0.1856, 0.2212]$
$[0.1773, 0.2122, 0.2522]$	$[0.1699, 0.2089, 0.2456]$
$[0.1306, 0.1639, 0.1988]$	$[0.1239, 0.1597, 0.1930]$
$[0.0818, 0.1107, 0.1378]$	$[0.0784, 0.1099, 0.1360]$

Table 8: In this table we report the set of quantiles obtained from the distribution of the multifractality proxy $\Delta H(1, 2)$ on the two tranches T_1 and T_2 for 1000 simulated time series whose hierarchical order $n_{T_1} = n_{T_2}$. Probabilities p's of the model are initialised to take values in $[0.4, 0.6]$. One observes an almost rigid shift towards smaller values of the distribution of the observed $\Delta H(1, 2)$s when the order decreases.

with corresponding risks

$$\Gamma_i \to K_{a_1}, \ldots, K_{a_{n_i}} \tag{4}$$

$$\Gamma_j \to K_{b_1}, \ldots, K_{b_{n_j}}. \tag{5}$$

Note that the Γ_i and Γ_j may or may not overlap. Denoting c the arbitrary node in the dendrogram, we consider also the sets

$$\begin{aligned} \Gamma_i \cap \Gamma_j &= \{c : c \in \Gamma_i \wedge c \in \Gamma_j\} \\ \Gamma_i \setminus \Gamma_j &= \{c : c \in \Gamma_i \wedge c \notin \Gamma_j\} \\ \Gamma_j \setminus \Gamma_i &= \{c : c \in \Gamma_j \wedge c \notin \Gamma_i\}. \end{aligned} \tag{6}$$

We drop the subscript t for the sake of readability and consider the two random variables r_i and r_j at arbitrary time t. The correlation coefficient between two returns r_i and r_j is given by

$$\rho_{ij} = \frac{\mathrm{Cov}(r_i r_j)}{\sqrt{\mathrm{Var}(r_i)\mathrm{Var}(r_j)}}. \tag{7}$$

The covariance between r_i and r_j is

$$\mathrm{Cov}(r_i r_j) = \langle r_i r_j \rangle = \langle \epsilon_i \epsilon_j \rangle \langle x_t^2 \rangle \langle Y_{n_i} Y_{n_j} \rangle \tag{8}$$

$$= \langle \epsilon_i \epsilon_j \rangle \langle x_t^2 \rangle \prod_{x : a_x \in \Gamma_i \cap \Gamma_j} \langle e^{2K_x} \rangle \prod_{l : a_l \in \Gamma_i \setminus \Gamma_j} \langle e^{K_l} \rangle \prod_{h : a_h \in \Gamma_j \setminus \Gamma_i} \langle e^{K_h} \rangle. \tag{9}$$

Since $\langle K_m \rangle = p_m$, we have $\langle e^{2K_m} \rangle = p_m(e^2 - 1) + 1 = \zeta_2(p_m)$ and $\langle e^{K_m} \rangle = p_m(e - 1) + 1 = \zeta_1(p_m)$. One also has

$$\mathrm{Var}(r_i) = \langle \epsilon_i^2 \rangle \prod_{a \in \Gamma_i} \langle e^{2K_a} \rangle = \langle \epsilon_i^2 \rangle \prod_{m=1}^{n_i} \langle e^{2K_m} \rangle \tag{10}$$

$$\mathrm{Var}(r_j) = \langle \epsilon_i^2 \rangle \prod_{b \in \Gamma_j} \langle e^{2K_b} \rangle = \langle \epsilon_i^2 \rangle \prod_{m=1}^{n_j} \langle e^{2K_m} \rangle. \tag{11}$$

The correlation coefficient therefore is

$$\rho_{ij} = \mathrm{Corr}(\epsilon_i \epsilon_j) \frac{\prod\limits_{x : a_x \in \Gamma_i \cap \Gamma_j} \zeta_2(p_x) \prod\limits_{l : a_l \in \Gamma_i \setminus \Gamma_j} \zeta_1(p_l) \prod\limits_{h : a_h \in \Gamma_j \setminus \Gamma_i} \zeta_1(p_h)}{\left(\prod\limits_{m=1}^{n_i} \zeta_2(p_m) \prod\limits_{q=1}^{n_j} \zeta_2(p_m) \right)^{1/2}}. \tag{12}$$

The last expression can be further simplified by noting that

$$\prod_{m=1}^{n_i} \zeta_2(p_m) = \prod_{z:a_x \in \Gamma_i \cap \Gamma_j} \zeta_2(p_x) \times \prod_{l:a_l \in \Gamma_i \setminus \Gamma_j} \zeta_2(p_l) \tag{13}$$

and

$$\prod_{m=1}^{n_j} \zeta_2(p_m) = \prod_{z:a_x \in \Gamma_i \cap \Gamma_j} \zeta_2(p_x) \times \prod_{h:a_h \in \Gamma_j \setminus \Gamma_i} \zeta_2(p_h). \tag{14}$$

The correlation coefficient ρ_{ij} then reads

$$\rho_{ij} = \mathrm{Corr}(\epsilon_i \epsilon_j) \frac{\prod_{x:a_x \in \Gamma_i \cap \Gamma_j} \zeta_2(p_x) \prod_{l:a_l \in \Gamma_i \setminus \Gamma_j} \zeta_1(p_l) \prod_{h:a_h \in \Gamma_j \setminus \Gamma_i} \zeta_1(p_h)}{\prod_{z:a_x \in \Gamma_i \cap \Gamma_j} \zeta_2(p_x) \left(\prod_{h:a_h \in \Gamma_j \setminus \Gamma_i} \zeta_2(p_h) \prod_{l:a_l \in \Gamma_i \setminus \Gamma_j} \zeta_2(p_l) \right)^{1/2}} \tag{15}$$

which yields

$$\rho_{ij} = \mathrm{Corr}(\epsilon_i \epsilon_j) \frac{\prod_{l:a_l \in \Gamma_i \setminus \Gamma_j} \zeta_1(p_l) \prod_{h:a_h \in \Gamma_j \setminus \Gamma_i} \zeta_1(p_h)}{\left(\prod_{h:a_h \in \Gamma_j \setminus \Gamma_i} \zeta_2(p_h) \prod_{l:a_l \in \Gamma_i \setminus \Gamma_j} \zeta_2(p_l) \right)^{1/2}}. \tag{16}$$

Defining

$$\mathcal{F}_{ij}(\mathbf{p}; \Gamma_i, \Gamma_j) = \frac{\prod_{l:a_l \in \Gamma_i \setminus \Gamma_j} \zeta_1(p_l) \prod_{h:a_h \in \Gamma_j \setminus \Gamma_i} \zeta_1(p_h)}{\left(\prod_{l:a_l \in \Gamma_i \setminus \Gamma_j} \zeta_2(p_l) \prod_{h:a_h \in \Gamma_j \setminus \Gamma_i} \zeta_2(p_h) \right)^{1/2}} \tag{17}$$

we finally have

$$\rho_{ij} = \mathrm{Corr}(\epsilon_i \epsilon_j) \mathcal{F}_{ij}(\mathbf{p}; \Gamma_i, \Gamma_j). \tag{18}$$

Some important remarks:

- The total correlation is factorized into two separate contributions, the first one associated with the multivariate Gaussian vector $\boldsymbol{\epsilon}_t$ and the second one only dependent on the probabilities of the nodes, that is on the hierarchy.

- The hierarchy factor $\mathcal{F}_{ij}(\mathbf{p}; \Gamma_i, \Gamma_j)$ is always smaller than one and thus acts as a perturbation of $\text{Corr}(\epsilon_i \epsilon_j)$, damping it according to the values assumed by the parameters p's.

- The two limit cases $p_m = 0$, $\forall m = 1, \ldots, N-1$ and $p_m = 1$, $\forall m = 1, \ldots, N-1$ both correspond to the hierarchy factor being 1. In both cases the effect of the hierarchy is null and one has $\rho_{i,j} = \text{Corr}(\epsilon_i, \epsilon_j)$.

Overall one can conclude that including a hierarchical structure in the volatility modelling introduces a perturbation to the standard multivariate model correlation matrix, which is nonetheless recovered when the probabilities of the internal risks are all null or all unity. These two cases correspond indeed to have the hierarchical structure disappearing as there wouldn't be any difference between different trees.
We show in Figure 14 the distribution of observed correlation coefficients on different realisations of DHM with probabilities taking values in different ranges. We can see how increasing the range of probability values shifts the bulk of the distribution to the right, which eventually matches the one of a log-normal multivariate model, where the hierarchical factors is turned off.

References

[1] N. Musmeci and al. in preparation. 2014.

[2] J.-P. Bouchaud and M. Potters. *Theory of financial risk and derivative pricing: from statistical physics to risk management.* Cambridge Univ Pr, 2003.

[3] Bradley Efron. Nonparametric estimates of standard error: The jackknife, the bootstrap and other methods. *Biometrika*, 68(3):589–599, 1981.

[4] Ulrich A Müller, Roland Bürgi, and Michel M Dacorogna. Bootstrapping the economy–a non-parametric method of generating consistent future scenarios. *MPRA Paper, University Library of Munich*, 2004.

[5] F. Pozzi, T. Di Matteo, and T. Aste. Exponential smoothing weighted correlations. *The European Physical Journal B*, 85:175, 2012.

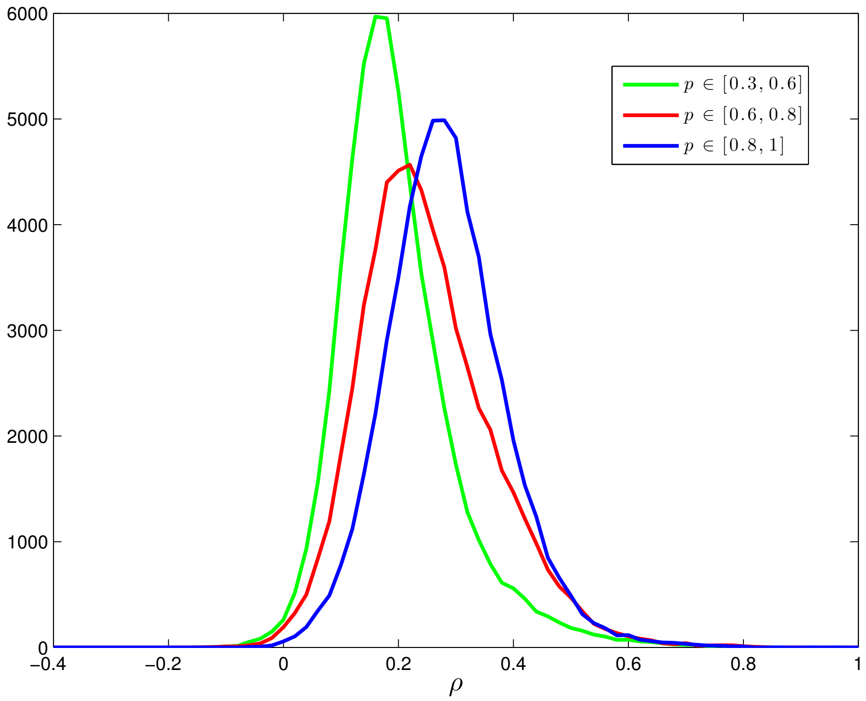

Figure 14: Distributions of the observed correlation coefficients on simulated DHM multivariate time series with probabilities of the internal risks in different ranges, reported in the legend.
